# Cost-effectiveness of prehabilitation of elderly frail or pre-frail patients prior to elective surgery (PRAEP-GO) versus usual care – Protocol for a health economic evaluation alongside a randomized controlled trial

**DOI:** 10.1186/s12877-024-04833-5

**Published:** 2024-03-06

**Authors:** Helene Eckhardt, Wilm Quentin, Julia Silzle, Reinhard Busse, Tanja Rombey

**Affiliations:** 1 Department of Health Care Management, Institute of Technology and Management, Technische Universität Berlin, Straße des 17. Juni 135, 10623 Berlin, Germany; 2 Planetary & Public Health, University of Bayreuth, Universitätsstraße 30, 95447 Bayreuth, Germany

**Keywords:** Health care economics, Health care evaluation mechanisms, Cost-effectiveness analysis, Trial-based health economic evaluation, Prehabilitation, Preoperative exercise, Frailty

## Abstract

**Background:**

Prehabilitation aims to improve patients' functional capacity before surgery to reduce perioperative complications, promote recovery and decrease probability of disability. The planned economic evaluation is performed alongside a large German multi-centre pragmatic, two-arm parallel-group, randomized controlled trial on prehabilitation for frail elderly patients before elective surgery compared to standard care (PRAEP-GO RCT). The aim is to determine the cost-effectiveness and cost-utility of prehabilitation for frail elderly before an elective surgery.

**Methods:**

The planned health economic evaluation comprises cost-effectiveness, and cost-utility analyses. Analyses are conducted in the German context from different perspectives including the payer perspective, i.e. the statutory health insurance, the societal perspective and the health care provider perspective. Data on outcomes and costs, are collected alongside the ongoing PRAEP-GO RCT. The trial population includes frail or pre-frail patients aged ≥70 years with planned elective surgery. The intervention consists of frailty screening (Fried phenotype), a shared decision-making conference determining modality (physiotherapy and unsupervised physical exercises, nutrition counselling, etc.) and setting (inpatient, day care, outpatient etc.) of a 3-week individual multimodal prehabilitation prior to surgery. The control group receives standard preoperative care.

Costs include the intervention costs, the costs of the index hospital stay for surgery, and health care resources consumed during a 12-month follow-up. Clinical effectiveness outcomes included in the economic evaluation are the level of care dependency, the degree of disability as measured by the WHO Disability Assessment Schedule 2.0 (WHODAS 2.0), quality-adjusted life years (QALY) derived from the EQ-5D-5L and the German utility set, and complications occurring during the index hospital stay. Each adopted perspective considers different types of costs and outcomes as outlined in the protocol. All analyses will feature Intention-To-Treat analysis. To explore methodological and parametric uncertainties, we will conduct probabilistic and deterministic sensitivity analyses. Subgroup analyses will be performed as secondary analyses.

**Discussion:**

The health economic evaluation will provide insights into the cost-effectiveness of prehabilitation in older frail populations, informing decision-making processes and contributing to the evidence base in this field. Potential limitation includes a highly heterogeneous trial population.

**Trial registration:**

PRAEP-GO RCT: NCT04418271; economic evaluation: OSF (https://osf.io/ecm74).

**Supplementary Information:**

The online version contains supplementary material available at 10.1186/s12877-024-04833-5.

## Introduction

### Background

A surgery represents an exceptional stress situation for patients’ body and mind, where the likelihood of unfavourable perioperative outcomes may depend on patient characteristics [1, 2]. In particular, frail patients, characterised by factors, such as diminished muscle strength, low walking speed, mental deterioration along with advanced age, tend to have a higher likelihood of experiencing worse health outcomes and higher resource utilisation such as discharge to a long-term facility or longer stay on the intensive care unit following surgery [3–7]. One concept of how to meet physical and mental weakness of frail elderly could be a (multimodal) prehabilitation prior to surgery [8].

Prehabilitation can be defined as a complex intervention that includes several therapeutic elements (modalities) and aims to prepare a patient for surgery or another stressor by increasing patient’s functional and cognitive capacity, and thereby increasing the patient’s resilience after surgery. Prehabilitation is often individualised for each patient and may include nutrition, physiotherapy, cognitive exercises, and other therapeutic elements. Due to a change in patient management, prehabilitation can be further characterised as a complex intervention.

Research on the clinical effectiveness of prehabilitation has shown that prehabilitation can have a significant impact on patients' postoperative outcomes. Patients who undergo prehabilitation are more likely to experience improvement in functional capacity [9, 10], a shorter hospital stay, fewer complications, and a faster recovery time [11]. However, the evidence on the effectiveness of prehabilitation in frail surgical patients of advanced age is sparse. Recent systematic reviews found that the main focus of current trials in frail surgical patients lies in the prevention of disability [12–14]. Positive effects were found on the reduction of the length of stay and complications, as well as on the improvement of post-surgical functional capacity.

Overall, the available body of evidence often focuses on cancer [9, 11, 15, 13, 16, 17] or orthopaedic surgeries [10, 18], and is predominantly of low quality. Moreover, recent systematic reviews did not identify any cost-effectiveness studies of prehabilitation in frail surgical patients of advanced age [14, 19].

Given the potential benefits, the increasing interest in the health care field, and the need for high-quality evidence and cost-effectiveness evaluations of prehabilitation for frail older patients prior to surgery, this protocol outlines a planned health economic evaluation of prehabilitation for this population prior to elective surgery in Germany. The economic evaluation will be conducted alongside a randomized controlled trial (PRAEP-GO RCT; NCT04418271) [20, 21].

### Aim and objectives

The planned health economic evaluation seeks to evaluate value for money of the complex intervention consisting of frailty screening, a shared decision-making conference and prehabilitation targeting frail or pre-frail elderly prior to elective surgery, compared to usual care, for the German health care system. The evaluation includes cost-effectiveness assessment from different perspectives. The aim is to provide advice to relevant stakeholders and decision-makers in the implementation of prehabilitation in routine health care in Germany, particularly in the context of statutory health insurance (SHI) coverage decisions. 

## Methods

The protocol of this evaluation was registered in OSF Registries on 29 June 2023 (https://osf.io/ecm74 [22]). The methods were guided by ISPOR recommendations on Good Research Practice in cost-effectiveness analysis alongside clinical trials [23] and by relevant textbooks [24]. Reporting of this protocol followed the Consolidated Health Economic Evaluation Reporting Standards (CHEERS 2022) [25]. Parts of the description of the study population and intervention are adapted from the publication of the trial protocol [20].

### The PRAEP-GO RCT

The PRAEP-GO RCT is an ongoing pragmatic, two-arm parallel-group, randomized, controlled, multicentre superiority trial in frail or pre-frail patients undergoing elective surgery in Germany with an allocation ratio of 1:1 per hospital and a follow-up period of 12 months postoperatively [20]. The PRAEP-GO trial was registered with ClinicalTrials.gov (NCT04418271) on 5 June 2020 [20, 21]. The investigators plan to recruite 1,400 trial participants over 3 years (enrolment period 30 June 2020 to 13 July 2023) [20, 21].

### Target population

The PRAEP-GO trial enrols frail or pre-frail patients undergoing elective surgery in Germany satisfying the following inclusion criteria [20, 21]: i) age ≥ 70 years, ii) planned elective surgery/interventional procedure, iii) expected duration of anaesthesia > 60 min, and iv) pre-frail or frail based on Fried’s frailty phenotype. Patients are defined as pre-frail with at least one item indicated as positive and as frail with three or more positive items of Fried’s frailty phenotype. Measurements of inclusion parameters and exclusion criteria are detailed elsewhere [20].

#### Subgroups

In the economic analyses, all patients included in the trial will be analysed. As the outcomes may differ depending on frailty status [26], the type and indication of the planned elective surgery (e.g. heart surgery, orthopaedic surgery, surgery for oncologic indication, etc.) and demographic variables, such as age and sex, subgroup analyses will be conducted given a sufficient size of a subgroup. For the planned subgroup analyses, the study population will be stratified by frailty status into a frail and pre-frail patient group according to the inclusion criteria. The type of planned elective surgery will be defined according to the main surgical diagnoses at baseline and divided into oncologic and non-oncologic indications, and by type of surgery (e.g., heart surgery, orthopaedic surgery, etc.).

### Setting and location

The ongoing PREAP-GO trial is located in Germany with the participating study and prehabilitation centres located in the federal states of Bavaria, Berlin, Brandenburg and Schleswig-Holstein [20, 27]. At the time of registration of the trial in June 2020, 23 centres participated in the trial [20]. The decision-making context is the German SHI, which consists of 96 SHI funds as of January 2023 and covers approximately 90% of the country's population [28]. Initially, trial participation was limited to patients of one SHI fund (BARMER). However, in December 2020, the trial participation was extended to patients insured with all other SHI funds in Germany [20], thereby increasing the representativeness of the study population. The intervention may be delivered in one of the four different settings: inpatient, day care, outpatient physiotherapy and rehabilitation centres, or at home via a mobile rehabilitation team [20, 21].

### Intervention and comparator

#### Control group

The *control group* will receive no intervention aside from the usual care provided as part of the perioperative management process. The type of usual care provided typically varies based on the specialty of the surgical procedure and the specific hospital where it will be performed. Each patient receives a pre-assessment by the responsible specialty and anaesthesiology a few days before surgery.

#### PRAEP-GO intervention

Patients in the *intervention group* will receive the PRAEP-GO intervention, a complex intervention comprising multiple elements: A) frailty screening, B) a shared decision-making (SDM) conference and C) a 3-week individualized multimodal prehabilitation program before elective surgery as described in Table 1.

**Table 1:** Description of the intervention

**Intervention**	**Description**
**A) Frailty screening**	**Evaluation of five criteria by a nurse and a physician:** muscle strength, walking speed, subjective fatigue, unintentional weight loss, physical activity [29] **0 criteria = robust, 1-2 criteria = pre-frail, 3-5 criteria = frail**
**B) Shared decision-making (SDM) conference**	**Online conference** consisting of interdisciplinary, interprofessional teams and the patient or relatives **using a three-talk model**: ** 1. choice talk:** identification of the willingness to participate in the decision-making process, and discussion of needs and priorities during prehabilitation ** 2.** **option talk:** patient or a proxy for the patient, multidisciplinary and multi-professional case conference (participants: anaesthesiology, geriatrics, and the respective field of the planned surgery or intervention, and either a therapist - physiotherapist or occupational therapist - or a nurse and a general practitioner) ** 3. decision talk:** definition of patient-centred goals for the prehabilitation period and establishment of a comprehensive prehabilitation plan, including a decision on the prehabilitation setting (inpatient, day clinic, outpatient^a^, home-based).
**C) Individualised prehabilitation program**	**Setting (where?):** inpatient, day clinic, ambulatory, or home-based as determined by the SDM **Intervention (what?):** supervised and unsupervised physical exercises; can include psychosocial and neurocognitive interventions, speech therapy, nutrition counselling, reduction of polypharmacy, and others. **Frequency and duration of intervention (how often and how long?):** Overall duration: 3-weeks, 45–48 total number of exercise sessions Session duration: 30 mins Frequency: • Supervised sessions: 5x/week, twice daily, which refers to a total of 30 supervised sessions • Unsupervised sessions: up to 6x/week, which refers to a total of up to 18 unsupervised sessions

Source: Own compilation based on [20]; Note: ^a^According to the definition of the Organization for Economic Co-operation and Development (OECD) [30]

##### Frailty screening

The frailty screening is performed by a nurse and a physician using the five criteria listed in Table 1, based on Fried's phenotype [29]. While the screening is conducted in both groups and serves as an inclusion criterion in the trial, the costs associated with the screening will only be considered in the intervention group, as frailty screening is not yet a standard of care in German hospitals.

##### Shared decision-making (SDM) conference

SDM implies the involvement of the patient in clinical decision-making. PRAEP-GO has adopted both interdisciplinary and interprofessional teams and is based on the “three-talk” model [31, 32] which consists of three phases – choice talk, option talk, and decision talk (Table 1). The SDM will take place after the baseline visit and after randomization. The goal of the SDM is to identify and discuss patient needs and priorities with the patient, and if the patient is willing to participate in further steps of the SDM, to decide in a multidisciplinary and multi-professional online conference about individual, patient-centred goals, along with the optimal setting for the prehabilitation. These goals are categorized into strength-, endurance-, mobility-, activities of daily living-, nutritional-related, and other interventions. Other goals of therapy are based on the identified needs and goals of the patient. Finally, a comprehensive prehabilitation plan, including a decision on the prehabilitation setting will be established. If the patient decides not to participate in the conference, the results of the conference will be discussed with the patient afterwards.

##### Prehabilitation

After completing the SDM process, participants in the intervention group will receive a 3-week prehabilitation program (Table 1) in one of the four different settings: inpatient, day care, outpatient physiotherapy and rehabilitation centres, or at home via a mobile rehabilitation team. All sessions of the prehabilitation program will be performed by multi-professional teams with special training in the defined prehabilitation program. During the 3-weeks of individual prehabilitation, 30 supervised sessions of multimodal therapy of 30 minutes each will be provided in the setting selected in the SDM conference, corresponding to 10 sessions per week –twice daily on five days per week. Additionally, patients are encouraged to do six unsupervised sessions per week, resulting in a total of 45–48 exercise sessions over the course of the prehabilitation period.

### Perspective of the economic evaluation

The trial-based health economic evaluation will be conducted from the three different perspectives, depending on the analysis type. We plan the economic evaluation considering multiple perspectives to enable an optimal societal decision [33], and to provide advice to the different relevant stakeholders and decision-makers in the implementation of prehabilitation in routine health care.

*Payer perspective*: The evaluation from the perspective of the SHI will consider direct medical costs of the intervention, as well as costs of the index hospital stay for surgery and resource utilisation during the 12-months-follow-up including formal long-term care.

*Societal perspective*: In addition to the direct medical costs incurred during the 12-months-follow-up, the evaluation from the societal perspective will also consider non-medical and indirect costs, such as opportunity costs of informal care or of the participation of relatives in the SDM, as well as user charges incurred by patients.

*Provider perspective*: From the perspective of health care providers, medical and non-medical costs of the intervention, as well as costs incurred during the index hospital stay for surgery until discharge, will be considered.

### Types of planned health economic evaluations

**Fig. 1:**
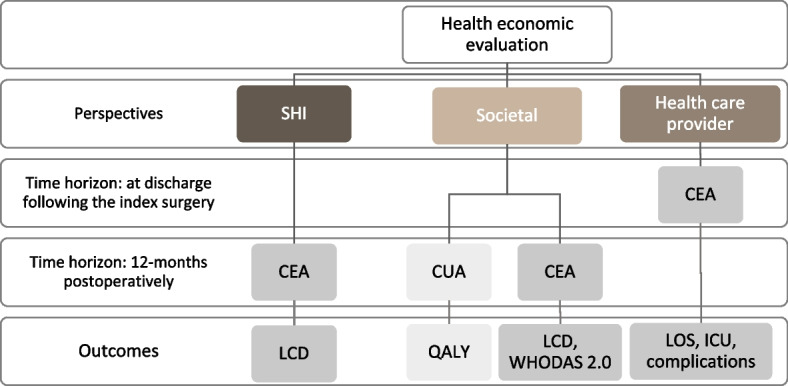
Overview of planned evaluations and outcomes. Abbreviations: CEA – cost-effectiveness analysis; CUA – cost-utility analysis; ICU – intensive care unit; LCD – Level of Care-Dependency; LOS – length of stay; QALY – Quality Adjusted Life Years; SHI – statutory health insurance; WHODAS – World Health Organization Disability Assessment Schedule. Note: Further details on the outcomes to be evaluated can be found in subsequent sections and Table 2

Figure 1 provides an overview of the types of evaluation planned depending on the evaluation perspective adopted. The analyses will involve the calculation of incremental cost-effectiveness ratios (ICER) and cost-utility ratios (ICUR). Both ICER and ICUR represent ratios of the difference in cost (incremental costs) between prehabilitation and usual care, divided by the difference in their effect on the outcome (incremental effect). A positive numerator in the ICER indicates higher costs in the intervention group. The interpretation of the sign of the denominator depends on the specific type of effect measure used and the direction of the positive or negative outcome. If a higher value of the effect measure is considered positive, a positive denominator suggests a positive outcome in favour of the intervention group. Conversely, if a lower value is considered positive, a positive denominator suggests a worse outcome in the intervention group. There are currently no cost-effectiveness or cost-utility thresholds defined in Germany [34]. The type of performed evaluation depends on outcomes being considered and on the evaluation perspective:

1. A cost-utility analysis (CUA) from a societal perspective, and
2. a cost-effectiveness analysis (CEA) from a societal perspective, the payer perspective (German statutory health insurance) and health care provider perspective.

### Time horizon and discount rate

The time horizons adopted differ depending on the economic perspective chosen. The time horizon from the SHI and the societal perspective is 12 months postoperatively after the index surgery. Extrapolation of costs and effects over a longer time period than one year would be associated with considerable uncertainties given the heterogeneous patient population receiving different types of planned surgeries and the population of frail and pre-frail elderly. From the provider perspective, the time horizon is until discharge following index surgery. Due to the follow-up period of a maximum of 12 months post-surgery, no discounting of costs and effects will be applied.

### Health outcomes

Selected outcomes, type of data collection and valuation of outcomes, as well as the relevant health economic perspectives, are presented in Table 2.

**Table 2:** Measurement and valuation of defined outcomes by perspective and type of analysis

**Outcome**	**Description**	**Unit**	**Data collection method**	**Valuation/ calculation**	**Health economic perspective**
QALY	Incremental Quality-Adjusted Life Year at 12 months	-0.661 to +1; -0.661=worst possible outcome +1=best possible health state	EQ-5D-5L questionnaire at 3-, 6-, 9-, and 12-months post-surgery; weighting of each measurement by 3/12	German Valuation Set [35]; death valued U=0; weighting of each U by 3/12	S
LCD	Probability of worsening of the Level of Care Dependency at least by one level between pre-intervention and 12 months post-surgery	1=worsening, 0=no change or improvement	Measured by the German New Assessment Tool of Care Dependency (NBI) by the research team of the study, the scale between 0 and 100 points is translated by rules of NBI into a 6 levels of care dependency: level 0 = no dependency, level 5 = full dependency	NBI [36]; 5=dead; difference in levels between 12 months and pre-intervention	S, SHI
WHO-DAS 2.0	Incremental degree of disability at 12 months	0-100; 100= full disability	WHO Disability Assessment Schedule questionnaire - WHODAS 2.0 12-items, in the past 30 days	WHODAS 2.0 [37]	S
LOS	Incremental length of hospital stay post-surgery	Days	Hospital billing dataset, § 301 para. 3 SGB V, § 21 KHEntgG, provided by hospitals	date of discharge - date of admission + 1	P
ICU	Incremental length of stay in ICU at discharge	Days	Hospital billing dataset, § 301 para. 3 SGB V, § 21 KHEntgG, provided by hospitals	as reported	P
COMP	Overall value of complications at discharge	0-100; 100=death	Secondary diagnoses, ICU stay, reported in hospital billing dataset, § 301 para. 3 SGB V, § 21 KHEntgG, provided by hospitals	Clavien-Dindo classification [38, 39], Comprehensive Complication Index [40–42]	P

*Abbreviations*: *COMP* Complications, *ICU* Intensive care unit, *LOS* Length of hospital stay, *KHEntgG* Hospital Remuneration Act (Krankenhausentgeltgesetz), *LCD* Level of Care Dependency, *S* societal perspective, *NBI* German New Assessment Tool of Care Dependency (das Neue Begutachtungsinstrument), *P* health care provider perspective, *QALY* Quality-Adjusted Life Year, *SGB V* Social Code Book 5 statutory health insurance (Sozialgesetzbuch - Fünftes Buch – Gesetzliche Krankenversicherung), *SHI* Statutory health insurance perspective, *WHODAS* World Health Organization Disability Assessment Schedule

#### Choice and measurement of health outcomes

The primary outcome from the societal perspective is the incremental quality adjusted life years (QALYs) at 12-months follow-up postoperatively. QALYs will be derived from the EQ-5D-5L questionnaire at 3-, 6-, 9-, and 12-months postoperatively and valuated by the German value set. According to the German value set for general German population, the best health state (11111) is valued with 1, death is valuated with 0 and the worst outcome (55555) is valuated with -0.661 [35]. All deaths will be identified at the end of the trial by querying the regional death registries. QALY was chosen for the health economic evaluation because a German valuation set is available [35, 43] and the use of the outcome type in the evaluation of prehabilitation is internationally recognised, as a recent systematic review has shown [19].

The primary outcome from the health care payer (German SHI) perspective is the "avoidance of worsening of the Level of Care-Dependency (LCD)”, which is a dichotomous variable derived from the ordinally scaled LCD with six levels, ranging from level 0 (no impairment of independence or no disability) to level 5 (most severe impairment with special needs for nursing care or most severe disability) measured at 12 months post-surgery and compared to the baseline.

The LCD will be measured and determined by the study personnel during a home visit using the German New Assessment Tool of Care Dependency (*Neues Begutachtungsinstrument, NBI*) [20]. The NBI assesses five categories: self-care, ability to self-manage the disease, ability to arrange daily life, mobility, and cognitive and communicative ability, resulting in a total maximum score between 0 and 100 points. These points are derived from the weighting of these categories, with the highest weighs assigned to self-care (40%) and ability to self-manage the disease (20%) [36]. The resulting sum of points can be translated into LCD scores: A person who is independent in all areas and not impaired in their abilities receives 0 points, while a person who experiences the greatest possible impairment in all areas of life, affecting their independence or abilities, is awarded 100 total points. A score of 12.5 points corresponds to LCD 1, 27 points to LCD 2, 47.5 points to LCD 3, 70 points to LCD 4, and 90 points to LCD 5. The requirements for LCD 5 are also met if someone has completely lost their gripping, standing, and walking functions - regardless of the score achieved in the six categories. In the context of the PRAEP-GO RCT, if a person dies, they are also assigned LCD 5.

The probability of worsening of LCD will be measured by comparing the patient status at 12 months with the baseline value: All patients experiencing deterioration in health status or independence, as measured by the NBI, i.e., those patients with a higher LCD compared to baseline, are assigned a score of 1. All other patients receive a score of 0. We decided to prioritize the deterioration of LCD as it represents the most undesirable outcome for both the patient and the payer, and one that should be prevented. LCD was selected as the primary outcome due to its considerable relevance to the German SHI perspective. For the payer, the instrument is an important measure of disability that is directly associated with long-term and short-term care expenses incurred by the SHI. From the societal perspective, the outcome will be analysed in secondary analyses.

From the health care provider perspective, incremental length of stay in the intensive care unit (ICU) measured as incremental days stayed in the ICU at discharge will be considered as primary outcome. Overall length of stay (LOS) and complications during the hospital stay will be considered as secondary outcomes. The ICU, LOS (LOS = Discharge date - Admission date +1) and complications based on secondary diagnoses will be reported by participating hospitals on the basis of billing data. The complications will be classified according to Clavien-Dindo classification [38, 39] or comprehensive complication index [40–42].

### Resource use and costs

#### Type of resource use and costs collected for health economic evaluation

**Fig. 2:**
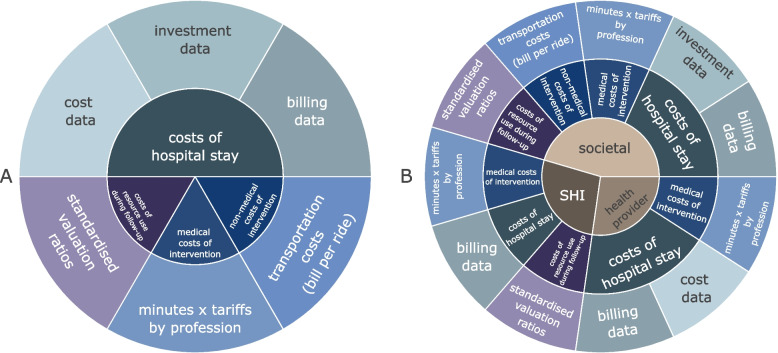
Overview of cost categories and respective types of data (**A**) used by adapted health economic perspective (**B**). Note: Detailed information on measurement and valuation of defined costs by perspective and type of analysis is available in the subsection “Measurement and valuation of resource use and costs” and in Additional file 1. Abbreviations: SHI – statutory health insurance

Cost categories and types of data used for the health economic evaluation are illustrated in Fig. 2A and B and described in more detail in the Additional file 1. The type of costs covered in the analysis depends on the adopted perspective (see Fig. 2B). While medical intervention costs are calculated in the same way for all perspectives (i.e., societal, SHI, and health care provider perspectives), the costs of hospital stay for surgery are calculated in different ways depending on the perspective. For instance, from the SHI perspective, only the hospital billing dataset, which represents the costs to the SHI, will be considered. From the societal perspective, additionally investment costs will be included into the analysis; from the provider perspective, for a set of hospitals with available data, the difference between actual cost data and billing data will be considered. The resource use during the 12-months follow-up begins on the first postoperative day and will be considered from the societal and SHI perspectives. On the contrary, opportunity costs of informal care and of the participation on the SDM will only be taken into account from the societal perspective. In the German SHI, patients are required to contribute to almost all services provided [44]. User charges of patients constitute 10% of the price of medical goods, such as pharmaceutical or auxiliary medical devices, with a minimum of 5 Euros and a maximum of 10 Euros. User charges for therapeutic services, nursing care at home or intensive care at home amount to 10% of costs and 10 Euros per prescription. The user charges for inpatient care and rehabilitation amount to 10 Euros per calendar day. The described user charges incurred by patients will be considered from the societal perspective while being subtracted from costs assessed from the SHI perspective.

#### Measurement and valuation of resource use and costs

Measurement and valuation of resource use and costs is described in detail in the Additional file 1. Both resource use and costs for the economic evaluation will be estimated using a bottom-up approach. The approach of data collection depends on the trial phase. The prehabilitation personnel collects and measures the resource use during the PRAEP-GO intervention. The costs and resource use during the 12 months follow-up is patient-reported, collected by study personnel via a questionnaire during a home visit, or via a telephone survey. Resource use will ultimately be valued by the health economists. Costs of hospital stay will be reported by participating hospitals. The level of aggregation of the resource use depends on the health care setting where health care is provided.

##### Costs of intervention

Estimation of the resource use during the intervention which was delivered in outpatient care is based on the duration of the intervention, the type of therapy delivered, and the type of health care professional involved according to the trial documentation. The resource use for prehabilitation will be valuated in average national prices of 2022 by the type of therapy, e.g., physiotherapy [45], nutrition therapy [46], occupational therapy [47]. To factor in the additional costs of the intervention delivered in the inpatient setting, overnight costs will be considered as well. Calculation of costs of frailty screening and SDM will be based on average wages in 2022 and time spent by profession. For prehabilitation delivered in an inpatient or day care setting, costs from outpatient care will be supplemented by the costs for overnight stay, infrastructure, material, staff, and administrative costs associated with the intervention according to the trial documentation. From the societal perspective, user charges incurred by patients for therapeutic services or inpatient care will be included in the overall cost assessment of the intervention.

##### Costs of index hospital stay

Data on inpatient stay for index surgery will be aggregated at the Diagnosis Related Group level (aG-DRG – German Diagnosis Related Groups with separated nursing staff costs) based on hospital billing data reported by the participating hospitals. These costs will be reported in prices of delivery year but will be transformed to a single price year (see below). To estimate inpatient costs from the societal perspective, user charges per patient and per calendar day will be added to the DRG costs.

##### Costs during follow-up

The resource utilisation reported by patients during the 12-months follow-up period will be collected as part of the clinical outcome collection using the Questionnaire for the Use of Medical and Non-Medical Services in Old Age (FIMA) [48]. The valuation of these resources will be determined using standardized valuation ratios in Euros [49], which are currently being updated based on 2020 prices. The standardized valuation ratios consider costs from the societal perspective and, consequently, include user charges for medical and therapeutic care, as well as (co-)payments for auxiliary medical devices incurred by patients. For the SHI perspective, the values can be adjusted based on underlying assumptions for the proportion of user charges, e.g. 15% for outpatient care [49]. Prices of pharmaceutical consumption will be calculated using the methods described by Braun et al. (2009) [50], in conjunction with the average annual fixed payment amounts, representing the maximum amounts that the SHI funds pay for this pharmaceutical drug, reported by the Federal Institute for Drugs and Medical Devices (BfArM) for the year 2022 [51].

#### Currency, price date, and conversion

All cost estimates from previous years will be converted into Euro 2022. The year 2022 was selected as it represents the year in which most patients had their index hospital stay, which likely presents the biggest cost block. Cost estimates will be adjusted for inflation using the average annual inflation rate in health care of 2.1% in 2022 in Germany [52]. Costs incurred in later years will be deflated accordingly using the annual average index reported by European Central Bank [53].

### Data management

Trained personnel at each participating study location collect study data by entering the data directly on-site into the Web electronic case report form (eCRF). Although paper-based case report forms (CRFs) are used as well, the data from these forms must be entered into the eCRF database at a later stage [20]. All the collected data will be managed using the research electronic data capture (REDCap) -database [54].

### Analytical methods

**Fig. 3:**
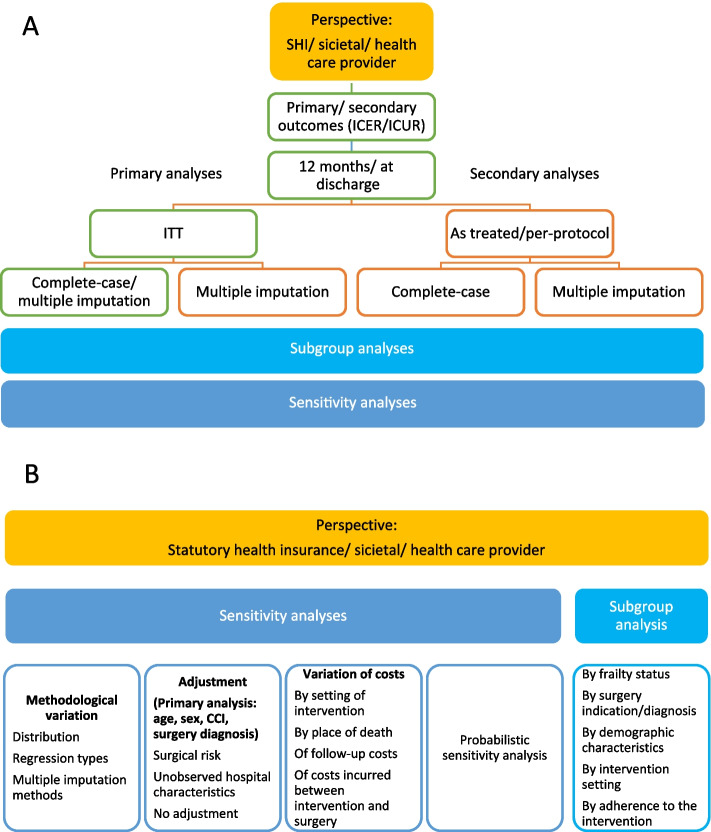
Schematic Representation of Planned (A) Primary and Secondary Analyses, (B) Subgroup Analyses, and Sensitivity Analyses. Abbreviations: CCI – Charlson Comorbidity Index; ICER – incremental cost effectiveness ratio; ICUR – incremental cost utility ratio; ITT – intention-to-treat; SHI – statutory health insurance

Figure 3 provides a summary of planned primary and secondary analyses (3A) as well as subgroup analyses, and sensitivity analyses (3B).

#### Adjustment for baseline characteristics

Due to the broad inclusion criteria of the pragmatic trial, i.e., different surgical indications resulting in different types of elective surgery, we anticipate that the study population may be highly imbalanced at baseline despite a well conducted random allocation of trial participants which usually should ensure a well-balanced distribution of observable and unobservable characteristics of study population between groups [55–57]. As baseline imbalance could introduce biases [58], we decided to apply adjustment for baseline age, sex, Charlson Comorbidity Index (CCI), surgery diagnosis as a proxy for the type of surgery and surgical risk estimate based on the type of surgery, as defined by the 2022 guideline from the European Society of Cardiology (ESC) [59], if standardised mean difference (SMD) in these variables is larger than 10%. SMD (Cohen’s d [60]) is defined as difference in means of a variable (e.g., age) between two groups divided by the pooled standard deviation from the mean of the variable among the participants of the two groups. The measure ranges between +/-1 to 0, with zero indicating perfect balance. SMD has been mostly used to evaluate the balance between groups in propensity score matching studies [61], but also in RCTs [62]. The CCI was selected as adjustment variable to account for health impairment. This selection is based on the recognition that CCI may be linked to costs and outcomes independently of any intervention [63], while it is not affected by the intervention itself. To account for unobserved characteristics of hospitals where patients were recruited and where the planned surgeries were performed, a dummy variable will be created for each hospital and incorporated into the analysis. The adjustment will be achieved by including the variables in the regression model and the results will be compared to the unadjusted model [57]. Baseline characteristics of the study population will be presented in tabularly format per group. Continuous (e.g. age) data will be presented in means and standard deviation or medians and interquartile ranges. Ordinal data will be presented as medians and interquartile ranges. Categorical data (e.g. sex) will be presented as percentages.

#### Handling missing data

After database closure in July 2024, available data will be assessed towards the amount of missing data, patterns of missingness and the type of missingness of data (i.e., missing completely at random, missing at random, missing not at random). As recommended by the German Institute of Quality and Efficiency in Health Care (IQWiG), if the proportion of study population not evaluated due to missing data is below 20%, the primary analyses will be conducted using complete case analysis [34]. Imputation methods will be employed in the secondary analysis. If the proportion of study population not evaluated due to missing at random data is 20% or higher, appropriate imputation methods will be employed in the primary analysis.

In both the primary and secondary analyses, the most appropriate solution for imputing missing data will be selected for each data type and outcome: Missing *demographic data* will be completed, where possible, using hospital billing data sets and administrative data of the BARMER SHI fund for trial participants insured with BARMER (see description of settings and location). The BARMER data will also be used for validation and completion of resource use and cost-data. If *CCI* is missing at baseline, the second available datapoint will be considered, as CCI is unlikely to considerably change within the observation period. If *EQ-5D-5L domains, WHODAS 2.0 or NBI domains* are missing at random, the missing values will be imputed using the Multivariate Imputation by Chained Equation (MICE) algorithm as suggested by [64, 65] using R packages “miceadds” and “mice”. Patterns of distribution of *cost and resource data* will also be evaluated and imputed by appropriate multiple imputation methods. The description of missing data and the applied methods of handling missing data will be reported as suggested in the literature [66–69]. The outcome and cost data at baseline and 12 months postoperatively will be presented depending on the data type. Continuous data will be presented in means and standard deviation or in medians and interquartile ranges. Ordinal data will be presented as medians and interquartile ranges. Categorical data will be presented as percentages. Effects will be presented in mean differences, or in odds ratios and 95% confidence intervals.

#### Analysis plan

##### Primary and secondary analyses

The primary analyses will be conducted from the societal and SHI perspectives taking into account relevant costs and incremental QALYs as well as the probability of worsening of Level of Care Dependency at 12 months. The ICUR and ICER will be calculated applying intention-to-treat principle (see Fig. 3A). The analysis will consider complete cases or imputed data, depending on the proportion of not evaluated study population due to missing data (see description above). Adjusted analysis employing the Seemingly Unrelated Regression (SUR) approach to model the relationship between costs and effects will be applied. The SUR enables simultaneous estimation of costs and effects and by doing so, adjusting for covariates and accounting for correlation between costs and effects. This approach allows for more efficient and precise estimates compared to separate regressions for costs and effects [70, 71, 62]. SUR was first described by Zellner (1962) [72] and suggested for application in health economic evaluations by Willan (2004) [70]. The intention-to-treat approach will encompass all randomised patients, irrespective of changes to their treatment plan, such as surgery being brought forward or cancelled, or cases where patients died either before the surgery, or before or during the prehabilitation intervention. Secondary analyses will consider further outcomes (e.g., WHODAS 2.0 from societal perspective), further perspectives (e.g., analysis of complications from the health care provider perspective), further imputation methods, as-treated and per-protocol analyses, unadjusted analyses and analysis of subgroups described in the section “Subgroups” and in Fig. 3B.

##### Sensitivity analyses

Sensitivity analyses to explore uncertainties of data will involve probabilistic and deterministic sensitivity analyses. To estimate the uncertainty surrounding the cost-effectiveness measures of primary analyses, non-parametric bootstrapping approach as described by Efron and Tibshirani (1993) [73] and probabilistic sensitivity analysis as suggested by Baio and Dawid (2015) [74] and Baio and Berardi (2017) [75] will be employed. Uncertainties surrounding costs will be explored using scenario analyses or one-way sensitivity analyses [76, 77]. Sensitivity analyses to explore methodological uncertainties will include variation of distribution of costs and effects, and adoption of regression analyses for different distribution types. For instance, as part of sensitivity analyses, the possible impact of the COVID-19 pandemic, associated restrictions and fear of infection will be assessed by considering the time between enrolment and surgery as well as adherence to prehabilitation as adjustment variables. These factors may have differed between the periods 2020/2021 and 2022/2023.

Results of conducted sensitivity analyses will be plotted in a cost-effectiveness plane per outcome. The probability of the PRAEP-GO intervention being cost-effective at different willingness-to-pay thresholds will be visualized as cost-effectiveness acceptability curves, derived from conducted sensitivity analyses [78, 79]. The maximum willingness-to-pay threshold considered will be 100,000 Euros per QALY gained and per unit of risk reduction in LCD. If the probability of the intervention being cost-effective does not reach 1 at the threshold of 100,000 Euros, the threshold will be further increased until the probability of 1 is reached. The threshold of 100,000 Euros was chosen arbitrarily for several reasons. First, in Germany, there is currently no specific threshold defined [34]. Second, it is above cost-effectiveness thresholds valid in other countries, such as England [80] or the Netherlands [81]. Third, it was used in other health economic evaluations, for instance, in the evaluation by Fernandes (2017) [82]. All analyses will be conducted in R using RStudio.

### Approach to engagement with different stakeholders

Clinicians actively participated in identifying the pertinent outcomes for the health economic evaluation. In addition, the implementation potential of prehabilitation as a new care model of preoperative care for frail elderly in Germany will be evaluated. This will involve active engagement from a diverse range of stakeholders, including clinicians, policymakers, administrators, and representatives from relevant patient and healthcare organizations. By incorporating the diverse perspectives of these stakeholders, the evaluation will capture a holistic understanding of the opportunities, challenges, and implications associated with the implementation of prehabilitation in German SHI. The protocol detailing the methods to be used in the evaluation of the implementation potential of the PRAEP-GO intervention was registered on 20 November 2023 [https://osf.io/ywezb [83]] and will be published separately.

### Trial status

The first patient was enrolled on 30 June 2020 immediately after the first COVID-19 wave in Germany. In the following months, enrolment had to be paused several times because hospitals did not perform non-urgent elective surgeries during further COVID-19 pandemic waves. Thus, the initial trial duration was extended until July 2024 [21], and the trial recruitment was extended from one to three years, which ended on 13 July 2023. All data analyses will only start after database closure in July 2024.

## Discussion

The current protocol outlines a trial-based health economic evaluation of prehabilitation in older frail elderly population prior to surgery, aiming to increase functional capacity, mitigate perioperative complications, promote recovery, and reduce the probability of disability. Internationally, this study represents one of the first large-scale RCTs and health economic evaluations conducted in a high-risk population.

Strengths of the planned health economic evaluation lie in the adoption of a societal perspective and considering resource use and costs from that perspective by collecting resource use during the follow-up and valuing it using standardized valuation ratios in Euro. However, there are some limitations that must be considered. First, the study population is likely to be very heterogeneous, which may introduce variability in the outcomes and complicate the interpretation of results. Second, the resource utilisation is being collected using a patient questionnaire over intervals of 3 months retrospectively which is prone to recall bias. Another important consideration is that the use of QALYs as a measure may not adequately reflect the preferences of older people and may poorly capture small health gains. This is due to the German utility set used in the valuation of EQ-5D-5L sets considers the general population, which may not fully represent the preferences and priorities of older patients [43]. Moreover, the study is not blinded, since it is not possible to blind trial participants to the intervention and there were no resources to effectively blind the outcome assessors. Additionally, the classification system of care levels (*Pflegegrade*) may not be as relevant to patients as for the SHI. Where possible, limitations will be addressed by sensitivity analyses applying scenario analyses.

Despite these limitations, the outlined health economic evaluation will provide valuable insights into the cost-effectiveness of prehabilitation in older frail populations, informing decision-making processes and contributing to the evidence base in this field. If the health economic evaluation demonstrates the superiority of prehabilitation, it could serve as a sustainable strategy for reducing costs and improving outcomes in particular in frail elderly population.

### Supplementary Information

**Supplementary Material 1.** 

## Data Availability

Not applicable.

## Abbreviations

aG-DRG: German Diagnosis Related Groups with separated (“a” stands for “ausgegliedert” = ”separated”) nursing staff costs
BfArM: Federal Institute for Drugs and Medical Devices (*Bundesinstitut für Arzneimittel und Medizinprodukte*)
CCI: Charlson Comorbidity Index
CEA: cost-effectiveness analysis
CHEERS: Consolidated Health Economic Evaluation Reporting Standards
CUA: cost-utility analysis
eCRF: electronical case report form
EQ-5D-5L: European Quality of Life 5 Dimensions 5 Level Version
FIMA: Questionnaire for the Use of Medical and Non-Medical Services in Old Age (*Fragebogen zur Inanspruchnahme medizinischer und nicht-medizinischer Versorgungsleistungen im Alter*)
G-BA: Federal Joint Committee (*Gemeinsamer Bundesausschuss*)
ICER: incremental cost effectiveness ratio
ICU: intensive care unit
ICUR: incremental cost utility ratio
IQWiG: Institute of Quality and Efficiency in Health Care (*Institut für Qualität und Wirtschaftlichkeit im Gesundheitswesen*)
LCD: Level of Care-Dependency
LOS: length of stay
MICE: Multivariate Imputation by Chained Equation
NBI: German New Assessment Tool of Care Dependency (*Neues Begutachtungsinstrument*)
PRAEP-GO: prehabilitation of elderly frail or pre-frail patients prior to elective surgery
QALY: Quality Adjusted Life Years
RCT: randomized controlled trial
REDCap: research electronic data capture
SHI: statutory health insurance
SMD: standardised mean difference
SUR: Seemingly Unrelated Regression
WHODAS: World Health Organization Disability Assessment Schedule
