# Supplementary material for: Cost-effectiveness of prehabilitation of elderly frail or pre-frail patients prior to elective surgery (PRAEP-GO) versus usual care – Protocol for a health economic evaluation alongside a randomized controlled trial
Source: BMC Geriatr. 2024 Mar 6;24:231. doi: 10.1186/s12877-024-04833-5 (PMC10916129; doi:10.1186/s12877-024-04833-5)
Supplement: Supplementary file 1 — Supplementary Material 1. [file 12877_2024_4833_MOESM1_ESM.pdf]

Additional file 1: Measurement and valuation of defined costs by perspective and type of analysis

Cost category	Description	Source of unit cost information (in Euro 2022)	Resource unit	Data collection	cost perspective
**Intervention**					
Frailty screening	Remuneration of frailty screening (application of the Fried's phenotype) by a nurse and/or physician in minutes + documentation lump sum per capita	Charité collective bargaining agreements for physicians (Ä2/3), nursing staff (P8/4), scientific staff (E13/3), provided by Charité	Minutes + capita	TD	S, SHI, P
SDM-conference participance by professionals	Remuneration of participating experts per profession and person	Charité collective bargaining agreements for physicians (Ä2/3), nursing staff (P8/4), scientific staff (E13/3), provided by Charité	Minutes	TD	S, SHI, P
Opportunity costs of participation in an SDM-conference by a family member	Opportunity costs of participation in an SDM-conference by a family member expressed in value of work given up	Standardised valuation ratios in Euro (Bock et al. 2015)*‡	Minutes	TD	S
Outpatient prehabilitation programme	Duration of therapy by the type of services and therapeutic area (physiotherapy, logopaedics, ergotherapy, nutritional counselling, speech therapy, etc)	Therapeutic prices by therapeutic area valid in 2022 available at GKV-SV-website (Startseite -> Krankenversicherung -> Ambulante Leistungen -> Heilmittel -> "relevant therapeutic area") according to § 125 (1) SGB V	Minutes	TD	S, SHI, P
Inpatient prehabilitation programme	Duration of therapy by the type of services and therapeutic area (physiotherapy, logopaedics, ergotherapy, nutritional counselling, speech therapy, etc), costs of inpatient formal care depending on the level of care dependency, infrastructure costs, material costs, physician costs, nursing care, function costs	Therapeutic prices by therapeutic area as described for "outpatient prehabilitation programme"; infrastructure, material, physician, and function costs as provided in trial documentation	Minutes + days	TD	S, SHI, P
Transportation	Transportation between intervention facility and patient's home	Bills, reported by hospitals	Ride	TD	S, SHI

Cost category	Description	Source of unit cost information (in Euro 2022)	Resource unit	Data collection	cost perspective
**Post-interventional hospital stay (inpatient or outpatient)**					
Hospital stay	Patient specific billing case data, including data on DRG, surgery and other procedures, diagnoses, length of stay	Hospital billing dataset, § 301 para. 3 SGB V, § 21 KHEntgG, provided by hospitals	DRG	HD	S, SHI, P
	Patient specific cost data†	provided by hospitals	DRG	HD	S, P
Investment costs	Estimation of investment costs per case	Investment valuation ratios for aG-DRG-version 2022 (available at InEK -> Investitionsbewertungsrelationen (IBR) -> IBR 2022) according to § 10 KHG	DRG	HD	S, P
**Medical resource use during 12 months of follow-up postoperatively**					
Therapeutic services	Therapeutic services by type of service (e.g. physiotherapy, speech therapy, etc.)	Standardised valuation ratios in Euro (Bock et al. 2015)*	Contacts	FIMA	S, SHI
Auxiliary aids by type	Auxiliary aids by type (e.g. rollator, ramp, etc.)	Standardised valuation ratios in Euro (Bock et al. 2015)*	Aid by type	FIMA	S, SHI
Day-surgery/outpatient surgery	Day-surgery/outpatient surgery by type of surgery	Standardised valuation ratios in Euro (Bock et al. 2015)*	Contacts or days	FIMA	S, SHI
User charges	(Co-) payments per prescription for drugs and devices or hospital charges per bed day	Standardised valuation ratios in Euro (Bock et al. 2015)*‡	not documented	FIMA	S
Pharmaceutical consumption	Pharmaceutical consumption based on reported active substance or brand, dose, and the number of units per day at 3, 6, 9 and 12 months, calculated for 3 months and translated in the largest available package size to cover the need	Average fixed payment amounts for non-innovative pharmaceuticals or patented pharmaceuticals without added benefit according to §§130a, 35 para 1 & 8 SGB V, valid in 2022 available at BfArM (Home-> Arzneimittel -> Arzneimittelinformationen -> Festbeträge und Zuzahlungen -> Arzneimittel-Festbeträge -> Festbeträge Archiv -> Festbeträge 2022). List prices of patented pharmaceuticals with added benefit without VAT. Calculation of payment from the SHI on basis of §130a SGB V also described by Braun et al. 2009.	Packages	FIMA	S, SHI

**Cost category**	**Description**	**Source of unit cost information (in Euro 2022)**	**Resource unit**	**Data collection**	**cost perspective**
Visits to providers of ambulatory health care (primary or secondary care)	Includes visits to doctors in primary and secondary specialised care, e.g. family doctor, internist, dentist, etc.	Standardised valuation ratios in Euro (Bock et al. 2015)*	Contacts	FIMA	S, SHI
Treatment in outpatient departments of hospitals	Outpatient treatment in hospital (e.g. consultation, emergency care, pre- and post-surgical care)	Standardised valuation ratios in Euro (Bock et al. 2015)*	Contacts	FIMA	S, SHI
Acute inpatient care (excluding psychiatric care)	Acute inpatient care (excluding psychiatric inpatient care) on normal ward and intensive care unit	Standardised valuation ratios in Euro (Bock et al. 2015)*	Days	FIMA	S, SHI
Psychiatric care	Psychiatric care in outpatient or inpatient facilities	Standardised valuation ratios in Euro (Bock et al. 2015)*	Contacts or days	FIMA	S, SHI
Rehabilitation	Rehabilitation in inpatient or outpatient facilities	Standardised valuation ratios in Euro (Bock et al. 2015)*	Contacts or days	FIMA	S, SHI
Formal care: ambulatory care or housekeeping	Ambulatory care or housekeeping in minutes	Standardised valuation ratios in Euro (Bock et al. 2015)*	Minutes	FIMA	S, SHI
Formal care depending on the level of care dependency: Day- or night-care	Care in day- or night-care facility by level of care dependency	Standardised valuation ratios in Euro (Bock et al. 2015)*	Days or nights	FIMA	S, SHI
Formal care depending on the level of care dependency: Short-time inpatient care	Care in short-time inpatient care facility by level of care dependency	Standardised valuation ratios in Euro (Bock et al. 2015)*	Days	FIMA	S, SHI
Opportunity costs of informal care or housekeeping by family members	Opportunity costs of informal care or housekeeping by a family member expressed in value of work given up	Standardised valuation ratios in Euro (Bock et al. 2015)*	Minutes	FIMA	S, SHI

Notes: † Cost data is only available from a subset of participating hospitals; *Currently, the standardized valuation ratios are being updated based on 2020 prices. In our analysis, we will use the updated valuation ratios based on 2020 prices; ‡ The standardized valuation ratios include values from a societal perspective, that take into account out-of-pocket expenses. This can lead to an overestimation of costs from the perspective of the statutory health insurance. **Abbreviations:** BfArM - Bundesinstitut für Arzneimittel und Medizinprodukte, Federal Institute for Drugs and Medical Devices; FIMA - Fragebogen zur Inanspruchnahme medizinischer und nicht-medizinischer Versorgungsleistungen im Alter, questionnaire for the use of medical and non-medical

**Cost category**	**Description**	**Source of unit cost information (in Euro 2022)**	**Resource unit**	**Data collection**	**cost perspective**
services in old age; GKV-SV - Spitzenverband Bund der Krankenkassen, National Association of Statutory Health Insurance Funds; HD - hospital documentation; S – societal perspective; P – health care provider perspective; SDM - shared decision making; SGB V - Sozialgesetzbuch 5, social code book 5; SHI - statutory health insurance; TD - trial documentation; VAT - value added tax					

Bock J-O, Brettschneider C, Seidl H, Bowles D, Holle R, Greiner W et al. Ermittlung standardisierter Bewertungssätze aus gesellschaftlicher Perspektive für die gesundheitsökonomische Evaluation. Gesundheitswesen 2015; 77(1):53–61.
